# Fluorescence Emission of Self‐assembling Amyloid‐like Peptides: Solution versus Solid State

**DOI:** 10.1002/cphc.202100570

**Published:** 2021-09-21

**Authors:** Carlo Diaferia, Chiara Schiattarella, Enrico Gallo, Bartolomeo Della Ventura, Giancarlo Morelli, Raffaele Velotta, Luigi Vitagliano, Antonella Accardo

**Affiliations:** ^1^ Department of Pharmacy and Research Centre on Bioactive Peptides (CIRPeB) University of Naples “Federico II” Via Mezzocannone 16 Naples 80134 Italy; ^2^ Institute of Applied Sciences and Intelligent Systems, CNR Via P. Castellino 111 Naples 80131 Italy; ^3^ IRCCS SDN Via Gianturco 113 Naples 80143 Italy; ^4^ Department of Physics “Ettore Pancini” University of Naples “Federico II” Via Cintia 26 Naples 80125 Italy; ^5^ Institute of Biostructures and Bioimaging (IBB), CNR Via Mezzocannone 16 80134 Naples Italy

**Keywords:** amyloid fluorescence, intrinsic fluorescence, peptide materials, self-assembly, solid state

## Abstract

Analysis of the intrinsic UV‐visible fluorescence exhibited by self‐assembling amyloid‐like peptides in solution and in solid the state highlights that their physical state has a profound impact on the optical properties. In the solid state, a linear dependence of the fluorescence emission peaks as a function of excitation wavelength is detected. On the contrary, an excitation‐independent emission is observed in solution. The present findings constitute a valuable benchmark for current and future explanations of the fluorescence emission by amyloids.

## Graphical abstract

**A matter of state**! The fluorescent behaviour of self‐assembling amyloid‐like peptides is dependent on the physical state of the sample. The detection of a linear dependence of the fluorescence emission peaks as a function of excitation wavelength occurs at solid state. Differently, in solution an excitation‐independent emission peak is observed.

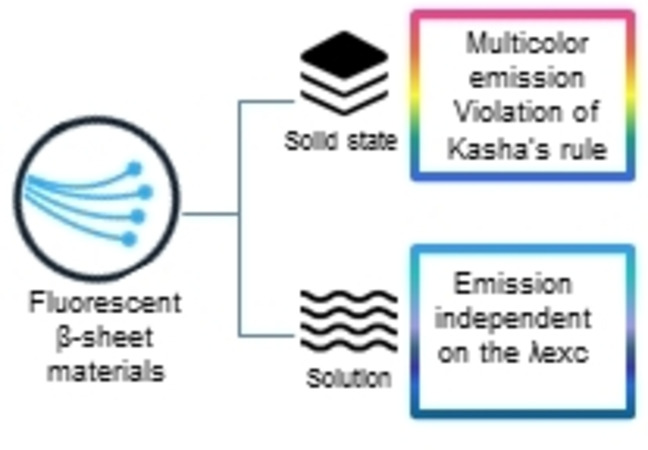

## 1 Introduction

Non‐covalent interactions play major roles in the activity of biomolecules as they dictate most of their physiological/pathological partnerships and oligomerizations. One of the areas in which non‐covalent interactions provide a crucial contribution is the amyloid‐like aggregation exhibited by an uncountable number of proteins and peptides.^[1]^ The ability of these biomolecules to form β‐structures is not restricted to natural proteins/peptides, but extends to a number of synthetic peptides whose study holds a remarkable technological importance.^[2]^ Indeed, peptide‐based nanostructures have progressively garnered significant research interest as innovative tools in biomedical, industrial and biotechnological areas, including tissue engineering,^[3]^ drug delivery,^[4]^ diagnostics,^[5]^ and antibacterial materials.^[6]^ Interestingly, amyloid‐like assemblies, in addition to their intricate structural organizations, also exhibit remarkable spectroscopic properties.^[7]^ In particular, studies carried out in the last two decades have demonstrated that amyloid‐like assemblies are endowed with the ability to emit visible fluorescence in a wide spectrum of possible wavelengths. The first experimental evidences of the special optical properties of amyloid‐like assemblies highlighted their ability to emit a blue‐green fluorescence upon excitation with near‐UV (370 nm) radiation.^[7a]^ Later studies have further shown the ability of these assemblies to absorb visible light and to emit green fluorescence.^[8]^ Intriguingly, recent additional scientific results have even enlarged the spectra of amyloid‐like emission to the red‐near Infrared range upon excitation at 670 nm.^[9]^ It is important to highlight that these photoluminescence (PL) phenomena have been detected also for peptides and polypeptides lacking aromatic amino acid residues (Phe, Tyr and Trp), typically believed to be responsible for the fluorescence emission in the far UV region.^[10]^ Despite the accumulation of experimental data, the origin of this fluorescence is still orphan of a clear and unique explanation.^[11]^ Possible physicochemical mechanisms underlying this phenomenon include: (i) electron delocalization through intra or intermolecular hydrogen bonds,^[8a]^ (ii) quantum confinement effects,^[12]^ (iii) charge transport through the H bond networks,^[13]^ (iv) carbonyl‐based autofluorescence,^[14]^ (v) nuclear quantum effect^[15]^ and (vi) distortion of the amide/carboxyl groups in the peptide bond.^[16]^ In this scenario, we have recently undertaken extensive structural and spectroscopic characterizations of self‐assembling oligopeptides formed by aromatic residues, with a particular attention to phenylalanine‐based peptides.[^8a^, ^17^] To investigate the properties of these peptides in solution we recently described the structural and the fluorescence properties of the hexaphenylalanine (F6) derivatized with polyethylene glycol (PEG) moieties of different lengths (PEG8‐F6, PEG12‐F6, PEG18‐F6, PEG24‐F6).^[8a]^ All of these polymer‐peptide conjugates showed a remarkable propensity to self‐assemble in β‐rich fibrillary nanomorphologies in water. As others amyloid‐like aggregates formed by many proteins/polypeptides,^[7]^ these self‐assembled nanosystems emit blue PL at 420–460 nm when excited in the wavelength interval 370–410 nm. Notably, PEG24‐F6, which contains PEG moiety with a molecular weight around 1300 Da, is able to emit green fluorescence with a maximum at ∼530 nm upon excitation of the sample at λ_exc_ ∼460 nm, a property that is rarely reported for self‐assembled proteins/peptide.^[8b]^ Interestingly, solid state films prepared for deposition of self‐assembled peptide solutions were successfully used for the fabrication of passive optical waveguides,^[18]^ thus suggesting wide applications in precision nanomedicine and integrated bio‐optics for these nanomaterials.

Considering the interest on the optical properties of self‐assembling peptides in the solid state and the scarce literature on the role that the physical state plays on their fluorescence emission, we herein report a comparative characterization of the fluorescence properties of different self‐assembling peptides in solution and at solid state. Initial analyses, conducted on the soluble PEGylated‐hexaphenylalanine PEG24‐F6, provided a clear evidence on the dependence of the fluorescence emission on the physical state of the assemblies. The generality of these results was evaluated through the characterization of a self‐assembling hexapeptide fragment of the Aβ peptide (Aβ_16‐21_) and of an unPEGylated F6 analogue in which both the termini are charged (H^+^‐F6‐O^−^).

## 2 Results and Discussion

### 2.1 Fluorescence Emission of PEG24‐F6

**Figure 1:**
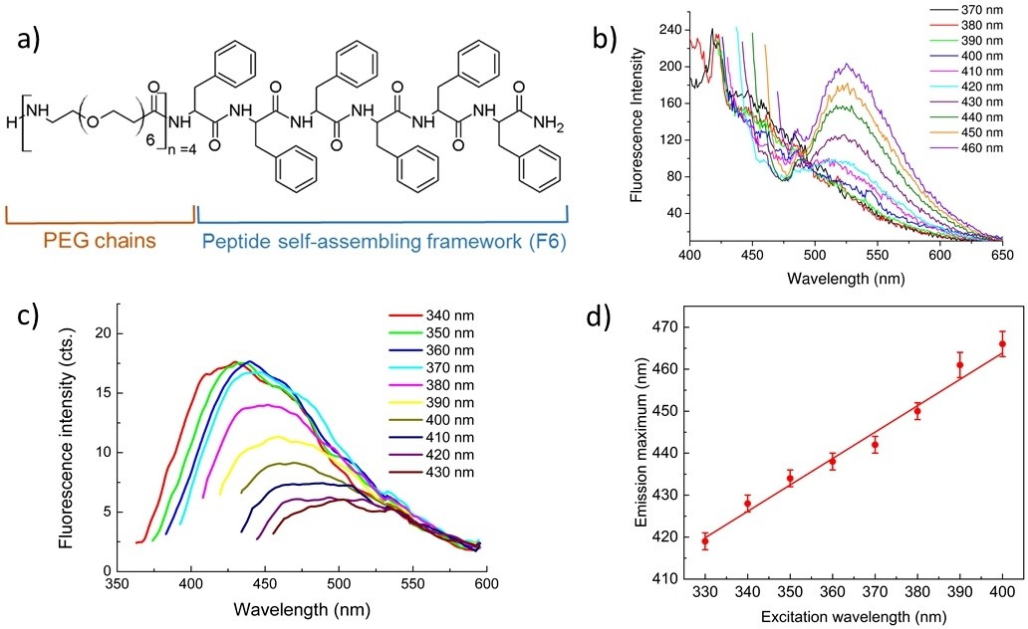
a) Schematic representation of polymer‐peptide derivative PEG24‐F6. b) Fluorescence emission spectra of a solution of PEG24‐F6 (at 10 mg/mL) upon excitation in the range 370<λ_exc_<460 nm. b) Fluorescence emission spectra of PEG24‐F6 film, drop‐casted from a solution at 10 mg/mL, upon excitation in the range 340<λ_exc_<430 nm. d) Plots of the maxima positions as function of the excitation wavelength for the measurements at the solid state. Therein, the linear best‐fit curve is also reported. The estimated values for slope and intercept were 0.63±0.04 and 212±13, respectively (Reduced χ^2^=1.045; Adj. R^2^=0.976).

The initial comparative analyses of the solution *versus* solid‐state fluorescence emission were conducted on the PEG24‐F6 polymer‐peptide (Figure 1a) whose structural characterization has been recently reported.^[8a]^ Despite its aromatic sequence, the peptide exhibits a good solubility in water (ca. 20.0 mg/mL). This solubility is given by the presence of PEG moiety on the N‐terminus of the peptide. Above a critical aggregate concentration of 6.2 μmol/L (13.9 μg/mL), the peptide self‐assembles in water in twisted fibrillary nanostructures, in which peptides are arranged in β‐sheet structures with an antiparallel orientation of β‐strands.^[8a]^ In line with our previous analysis, in solution the PEG24‐F6 amyloid‐like assemblies emit fluorescence in the green region of the visible spectrum with a peak at 525 nm that is independent of the excitation wavelength upon excitation with visible light (Figure 1b).^[8a]^ On the contrary, the fluorescence emission of the peptide film is radically different as it presents a variation of the maxima wavelength of the emitted fluorescence upon change of the excitation wavelength (Figure 1c). Such emission is linearly dependent on the excitation wavelength in the entire investigated range (Figure 1d). Although multicolor emissions has been previously reported by Rosenman and coworkers,^[19]^ the present findings represent the first evidence of a clear linear correlation between excitation/emission wavelengths for a self‐assembling peptide. A similar correlation between excitation/emission has been hitherto reported only for aggregates formed by the insulin and β‐lactoglobulin proteins.^[20]^

The comparison of the fluorescence spectra in Figure 1b and c shows that the highest peak detected in the solid‐state spectrum (∼525 nm) corresponds to the emission peak of the sample in solution. This evidence strongly indicates that the different spectroscopic behavior of these assemblies in solution and at the solid state is a manifestation of the so‐called red edge excitation shift (REES).^[21]^ The genesis of the REES effect arises from the interactions between a fluorophore and the surrounding context (solvent or local molecular matrix) in the ground and excited states.^[22]^ In a fully solvated environment, the fluorescence lifetime is much longer than the lifetime of the environmental relaxation. This allows a reorganization of the solvent around the excited fluorophore, thus generating an emission wavelength that is independent of the excitation wavelength. On the contrary, if the fluorescence lifetime is shorter than the lifetime of environmental relaxation, as observed in contexts with highly restricted mobilities, a wavelength‐dependent fluorescence emission is observed. The fluorescence data of PEG24‐F6 here reported perfectly fit into this an interpretative framework as we observe a single peak emission for the solvated assemblies whereas a wavelength‐dependent emission is observed in the rigid context of the solid‐state.

### 2.2 Fluorescence Characterization of Aβ_16‐21_

**Figure 2:**
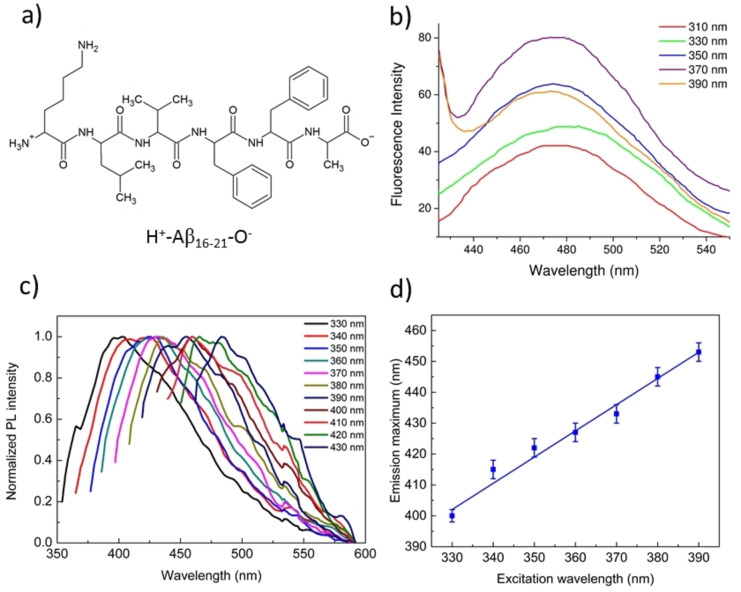
a) Chemical structure of H^+^‐Aβ_16‐21_‐O^−^. b) Fluorescence emission spectra of Aβ‐peptide from a solution at 10 mg/mL, upon excitation in the range 310<λ_exc_<390 nm. c) Normalized fluorescence spectra of the peptide film versus the excitation wavelength in the range between 330 and 430 nm. d) Plots of the maxima positions as function of the excitation wavelength at 5.0 mg/mL together with the corresponding best‐fit line. The estimated values for slope and intercept were 0.85±0.05 and 122±18, respectively (Reduced χ^2^=1.060; Adj. R^2^=0.979).

The fluorescence characterization described above for the self‐assembled PEG24‐F6 in solution and at the solid state suggests that the physical state of the sample can deeply affect its photoemissive behavior. In order to evaluate if these differences may be a general property of amyloid‐like assemblies independently of the size and physicochemical properties of their polypeptide chain, we extended out solution *versus* solid‐state fluorescence analyses to the self‐aggregating Aβ_16‐21_ peptide, which, in contrast to PEG24‐F6, does not present a repetitive peptide sequence (Figure 2a).^[8a]^

As expected, the structural characterization on Aβ_16‐21_ variant (SEM, Congo Red and Thioflavin T assay) confirmed its capability to self‐organize in β‐sheets rich nanostructures (see Figure S1 in the Supporting Information). The analysis of the photoluminescence properties of this peptide in solution evidences an intrinsic fluorescence with a maximum at 475 nm (Figure 2b). The wavelength of this emission peak is similar to that exhibited by Aβ_1‐42_ fibrils,^[13]^ but significantly different from that exhibited by PEG24‐F6 that presents the emission peak at 525 nm. Nevertheless, the wavelength of the emission peak is still independent on the wavelength of the excitation radiation.

To compare the solution *versus* solid‐state fluorescence properties of the assemblies formed by the Aβ_16‐21_ peptide, as for PEG24‐F6, we prepared solid aggregates from 10 μL peptide solutions at 5.0 mg/mL in H_2_O and 100 mg/mL in HFIP. These solutions were drop‐casted onto microscope glass slides and allowed to dry in air at room temperature. Photoemissive behavior of both the samples is reported in Figure 2c and in Figure S2 for 5 and 100 mg/mL, respectively. In perfect accordance with what observed for the PEGylated‐F6 peptide described above, the wavelength of the peak of the solid‐state fluorescence emission of Aβ_16‐21_ strongly depends on the excitation wavelength (Figure 2c), showing an evident linear behavior (Figure 2d). Again, the highest emission wavelength (∼480 nm) is nearly coincident with the single wavelength emission observed in solution. Moreover, as for PEG24‐F6, the wavelength of maximum emission is linearly dependent on the wavelength of the excitation radiation (Figure 2c). As a whole, the data collected on PEG24‐F6 and on the Aβ_16‐21_ peptide indicate the occurrence of the REES effect for the solvated systems and the excitation‐dependent PL emission at solid‐state.

### 2.3 Solid‐State Characterization of H^+^‐F6‐O^−^

**Figure 3:**
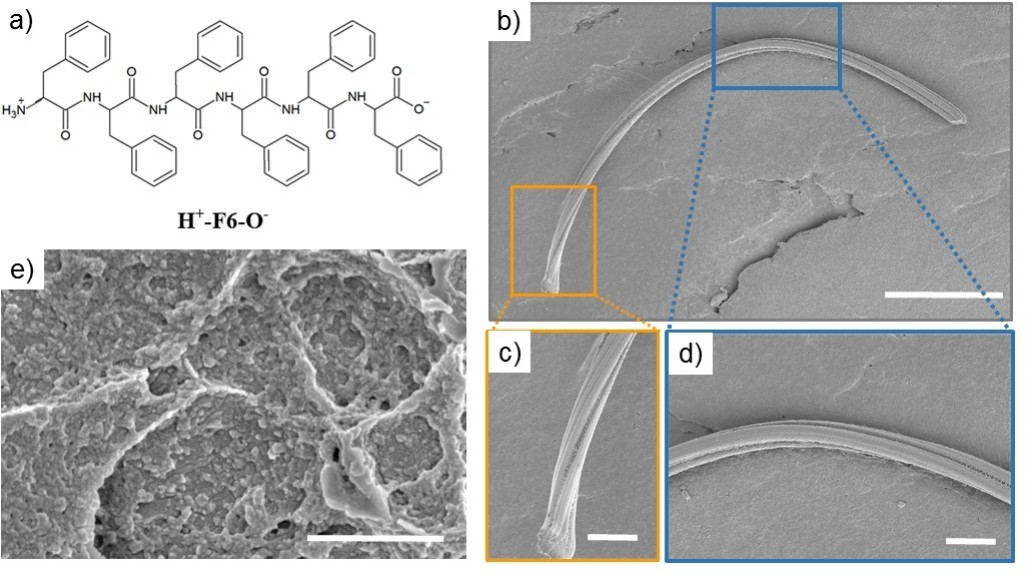
a) Schematic representation of the zwitterionic peptide H^+^‐F6‐O^−^. Selected scanning electron micrographs at 5.0 (b, c, d) and at 50 mg/mL (e). Scale bars are: 100 μm for (b), 20 μm for (c) and (d), and 1 μm for (e).

The excitation‐dependent PL emission observed for the solid‐state aggregates of PEG24‐F6 and Aβ_16‐21_ was further explored in the hexaphenylalanine peptide H^+^‐F6‐O^−^ **(**Figure 3a), which is the unPEGylated version of PEG24‐F6. The structural properties of this peptide were previously investigated by our group in comparison with its variants with charged/uncharged states of their C‐ and N‐termini.^[17b]^ Due to the lack of the PEG moiety the peptide is scarcely soluble in water; hence its photoemissive characterization was performed only at the solid state. The peptide solution was prepared in HFIP (1,1,1,3,3,3‐hexafluoro‐2‐propanol) that is a good solvent/co‐solvent for high hydrophobic Phe‐containing peptides.^[23]^ Air‐dried samples of the peptide for solid‐state experiments were prepared by drop‐casting peptide solutions at two different concentrations: 5.0 mg/mL and 50 mg/mL. Before starting with the spectroscopic characterizations, we preliminarily evaluated the morphology of the peptide nanostructures by Scanning Electron Microscopy (SEM). SEM micrographs, reported in Figure 3b–e, show a dependence of the morphology on the concentration. Indeed, at low concentration (5.0 mg/mL) the peptide assembles in twisted structures with a length and a thickness ranged between 300–1000 μm and 5–20 μm, respectively. On the other hand, at high concentration (50 mg/mL) SEM micrographs show a sort of qualitatively homogeneous film composed by aggregated structures distributed along all the directions with an apparently lower order degree (Figure 3e). This result suggests that there is an effect of the peptide concentration on its structural organization at the solid state. A similar effect was previously observed also for amyloid‐like peptide or protein at high concentration of salt.^[24]^ Air‐dried H^+^‐F6‐O^−^ samples were qualitatively characterized *via* fluorescence microscopy at different excitation wavelengths. As shown in Figure S3, peptide structures emit blue and green fluorescence independently of their morphologies (fibers or films).

**Figure 4:**
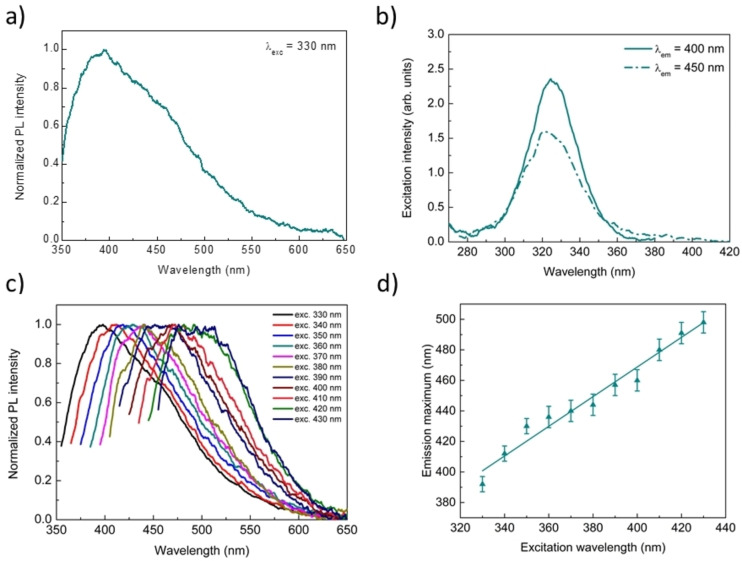
a) Normalized fluorescence spectrum of the peptide at λ_exc_=330 nm. b) Excitation spectra of the sample at 5.0 mg/mL setting λ_em_=400 nm (solid lines) and λ_em_=450 nm (dash‐dotted lines). c) Normalized fluorescence spectra of the peptide film versus the excitation wavelength in the range between 330 and 430 nm. d) Plots of the maxima positions as function of the excitation wavelength. Analogously to the previous samples, the best‐fit line is also depicted. The estimated values for slope and intercept were 0.97±0.06 and 80±23, respectively (Reduced χ^2^=1.135; Adj. R^2^=0.962).

A quantitative evaluation of the fluorescence emission(s) of this peptide at solid state indicate that H^+^‐F6‐O^−^ presents a maximum of the fluorescence emission at ∼400 nm and a shoulder at ∼450 nm upon excitation at 330 nm (Figure 4a). The excitation spectra of the same sample at 400 (continuous line) and 450 nm emission wavelength (dash‐dotted line) are reported in Figure 4b.

This fluorescence emission cannot be ascribed to the stacking of the aromatic side chains of the Phe residues as these interactions usually lead to an excimeric emission around 310–350 nm.[^17e^, ^25^] Rather, this fluorescence can be attributed to the β‐rich structure formed by the peptide.[^18^, ^26^] Analogously to the Aβ_16‐21_ peptide fragment and to PEG24‐F6, also H^+^‐F6‐O^−^ films show a multicolor emission profile as evidenced by the normalized fluorescence spectra *versus* excitation wavelength in the range from 330 and 430 nm (Figure 4c). Again, a linear correlation between the emission and the excitation wavelengths was observed (Figure 4d). Although the low solubility of this peptide prevented solution studies, these observations clearly indicate that the excitation‐dependent PL emission for hexaphenylalanine peptides is independent of the presence of the PEG moiety.

## 3 Conclusions

To the best of our knowledge, the comparative analyses of the solution *versus* solid‐state fluorescence here reported for PEG24‐F6 and Aβ_16‐21_ provide the first evidence that the physical state of the sample has a profound impact on the optical properties exhibited by peptides that self‐assemble in amyloid‐like structure. It is important to note that a similar behavior is detected for amyloid‐like systems that have distinct fluorescence properties in terms of the peak of the fluorescence emission in solution (475 nm for Aβ_16‐21_ and 525 for PEG24‐F6). Collectively, the reported results, along with previous evidence collected on the proteins insulin and β‐lactoglobulin^[20]^ and on thermally‐induced peptide aggregates,^[19]^ strongly indicate that the dependence of the emission wavelength from the excitation wavelength may be a general property of amyloid‐like systems. Moreover, they also highlight remarkable differences between solution and solid‐state fluorescence. Since these behaviors are strongly dependent on interaction of the fluorophore with the solvent and on the local context dynamics,^[27]^ the present findings may also provide insights into the hydration of amyloid‐like systems. In this respect, we have recently shown that the PEGylated Phe‐Phe dipeptides show a dependence of the emission wavelength from the excitation one in solution,^[28]^ thus indicating a different solvent distribution in these systems compared to those (Aβ_16‐21_ and PEG24‐F6) here investigated. Altogether, these data indicate that even similar amyloid‐like systems interact with the solvent in different ways. The investigation of differences and analogies in the PL properties of self‐assembling peptides/proteins at different physical states may represent a valuable tool for better characterizing these important and intricate supramolecular assemblies. Moreover, the interpretation of present data represents an important benchmark for current[^13^, ^16^] and future explanations of the fluorescence emission by amyloids.

## Experimental Section

### Materials

Protected N^α^‐Fmoc‐Phe‐OH, preloaded Fmoc‐Phe‐Wang resin, Rink amide MBHA (4‐methylbenzhydrylamine) and all reagents for coupling reactions, commercially available from Calbiochem‐Novabiochem (Laufelfingen, Switzerland), were used. All other chemicals materials, commercially available by Sigma‐Aldrich (Milan, Italy), Fluka (Bucks, Switzerland) or LabScan (Stillorgan, Dublin, Ireland) were used as received unless otherwise stated.

### Peptide Synthesis

H^+^‐F6‐O^−^ and PEG24‐F6 peptides were synthesized according to the method previously described.^[29]^ H^+^‐Aβ_16‐21_‐O^−^ was synthesized using the standard solid‐phase 9‐fluorenylmethoxycarbonyl (Fmoc) procedures. The Wang resin preloaded with Ala (substitution 0.60 mmol/g, 010 mmol) was used as solid phase support. The synthesis was carried out using a mixture of N,N‐dimethylformamide/N‐methyl‐2‐pyrrolidone (DMF/NMP, 1 : 1, *v/v*) as solvent phase. Before starting the peptide elongation, resins were swelled for 30 min in solvent medium. Fmoc deprotection was performed twice (each treatment for 10 min) using 30 % (*v/v*) piperidine in DMF/NMP. The Fmoc‐amino acid couplings were achieved by adding 2‐fold molar excess of Fmoc‐aa‐OH, mixed with equimolar amounts of 1‐hydroxybenzotriazole (HOBt), benzotriazol‐1‐yl‐oxy‐tris‐pyrrolidino‐phosphonium (PyBop) and 4‐fold molar excess of diisopropylethylamine (DIPEA). All couplings were performed twice for 40 minutes. Crude peptides were fully cleaved in acidic condition by TFA (trifluoroacetic acid)/H_2_O/TIS (triisopropylsilane) (90/5/5 *v/v/v*) mixture at room temperature for 2 hours. Then, peptides were precipitated with ice‐cold ether and lyophilized. Purity and chemical identity of the synthetic products were assessed by analytical LC‐MS analyses by using Finnigan Surveyor MSQ single quadrupole electrospray ionization (Finnigan/Thermo Electron Corporation San Jose, CA) using a column C18‐Phenomenex eluted with an H_2_O/0.1 % TFA (A) and CH_3_CN/0.1 % TFA (B) from 5 % to 70 % over 20 minutes at 200 μL/min flow rate.

H^+^‐Aβ_16‐21_‐O^−^ characterization: t_R_=15.00 min, MS (ESI^+^): m/z 723.90 calcd. for C_38_H_57_N_7_O_7_: [M+H^+^]=724.90.

### Sample Preparation

PEG24‐F6 solution was prepared by dissolving the lyophilized powder in double distilled water sonicating the sample for 1 minutes. Then peptide concentration was determined with UV‐Vis Thermo Fisher Scientific Inc (Wilmington, Delaware USA) Nanodrop 2000c spectrophotometer equipped with a 1.0 cm quartz cuvette (Hellma) using a molar absorptivity (ϵ_257_) of 1170 M^−1^ cm^−1^. The peptide solution was diluted at 10 mg/mL. Solutions of H^+^‐Aβ_16‐21_‐O^−^ at 5.0 and 100 mg/mL were prepared in water or in HFIP, respectively. The peptide concentration in water was assessed by UV‐Vis spectroscopy using of 390 M^−1^⋅cm^−1^ as molar absorptivity at 257 nm. Finally, stock solution of H^+^‐F6‐O^−^ was prepared dissolving the peptide powder directly in 1,1,1,3,3,3‐hexafluoro‐2‐propanol (HFIP) at 50 mg/mL. Subsequently, this solution was also diluted with HFIP to obtain a concentration of 5.0 mg/mL. For solid state characterization, these peptide solutions were drop‐casted onto flat microscope glass slides and allowed to air dry at room temperature.

### Congo Red (CR) Spectroscopic and Birefringence Assays

A freshly prepared stock solution of CR (7.0 mg/mL) in 10 mM phosphate buffer at pH 7.4 was filtered through 0.22 μm syringe and then diluted in water at 5.0 μM final concentration. This solution was incubated for 30 minutes at room temperature with 0.5 mg/mL of Aβ_16‐21_ peptide. After incubation, UV‐Vis spectra of Congo Red (CR) alone or incubated with Aβ_16‐21_ were recorded between 400 and 750 nm on Nanodrop 2000c spectrophotometer equipped with a 1.0 cm quartz cuvette (Hellma).

### Thioflavin T (ThT) Assay

Aggregate formation of H^+^‐Aβ_16‐21_‐O^−^ was assessed by fluorescence spectroscopy using ThT. This dye associates rapidly with β‐rich aggregates giving rise to an enhanced fluorescence emission at 482 nm.^[30]^ Spectra of an aqueous solution of 25 μM ThT, before and after the addition of Aβ‐derivative (5.0 mg/mL), were recorded at room temperature. The spectrum of the peptide solution alone was also acquired as reference. Fluorescence measurements were recorded between 460 and 650 nm exciting the samples at 450 nm.

### Scanning Electron Microscopy (SEM)

Morphological analysis of the nanostructures was carried out using field emission scanning electron microscope (Nova NanoSem 450‐FEI). Briefly, 10 μL of sample solutions of H^+^‐F6‐O^−^ (at 5.0 and 50 mg/mL) and of H^+^‐Aβ_16‐21_‐O^−^ (at 5.0 mg/mL) were dropped off on aluminum stub using a graphite adhesive tape. A thin coat of Au and Pd was sputtered at a current of 20 mA for 120 s. The sputter coated samples were then introduced into the specimen chamber and the images were acquired at an accelerating voltage of 2–5 kV, spot 3, through the Everhart Thornley Detector (ETD) and the Through the Lens Detector (TLD).

### Fluorescence Microscopy

10 μL of each peptide solution were drop‐casted on a clean coverslip glass, dried and imaged with fluorescence microscopy. Immunofluorescence images were taken with a Leica DM6M fluorescence microscope equipped with DFC 7000 T camera, at excitation wavelengths between 365 nm and 470 nm.

### Fluorescence Measurements

The samples, as above prepared, were spectrally characterized using a Jasco FP‐8300 spectrofluorometer equipped with an ILF‐835 integrating sphere. Fluorescence spectra of the films were acquired in a window up to 650 nm, probing the 330–430 nm excitation range at 10 nm steps. All acquisitions were averaged over five measurements after spectral correction.

## Conflict of interest

The authors declare no conflict of interest.

## Supporting information

As a service to our authors and readers, this journal provides supporting information supplied by the authors. Such materials are peer reviewed and may be re‐organized for online delivery, but are not copy‐edited or typeset. Technical support issues arising from supporting information (other than missing files) should be addressed to the authors.

Supporting Information
