# Supplementary material for: Fluorescence Emission of Self‐assembling Amyloid‐like Peptides: Solution versus Solid State
Source: Chemphyschem. 2021 Sep 21;22(21):2215–21. doi: 10.1002/cphc.202100570 (PMC8597038; doi:10.1002/cphc.202100570)
Supplement: Supplementary file 1 — Supporting Information [file CPHC-22-2215-s001.pdf]

ChemPhysChem

Supporting Information

Fluorescence Emission of Self-assembling Amyloid-like Peptides: Solution versus Solid State

Carlo Diaferia, Chiara Schiattarella, Enrico Gallo, Bartolomeo Della Ventura, Giancarlo Morelli, Raffaele Velotta, Luigi Vitagliano,* and Antonella Accardo*

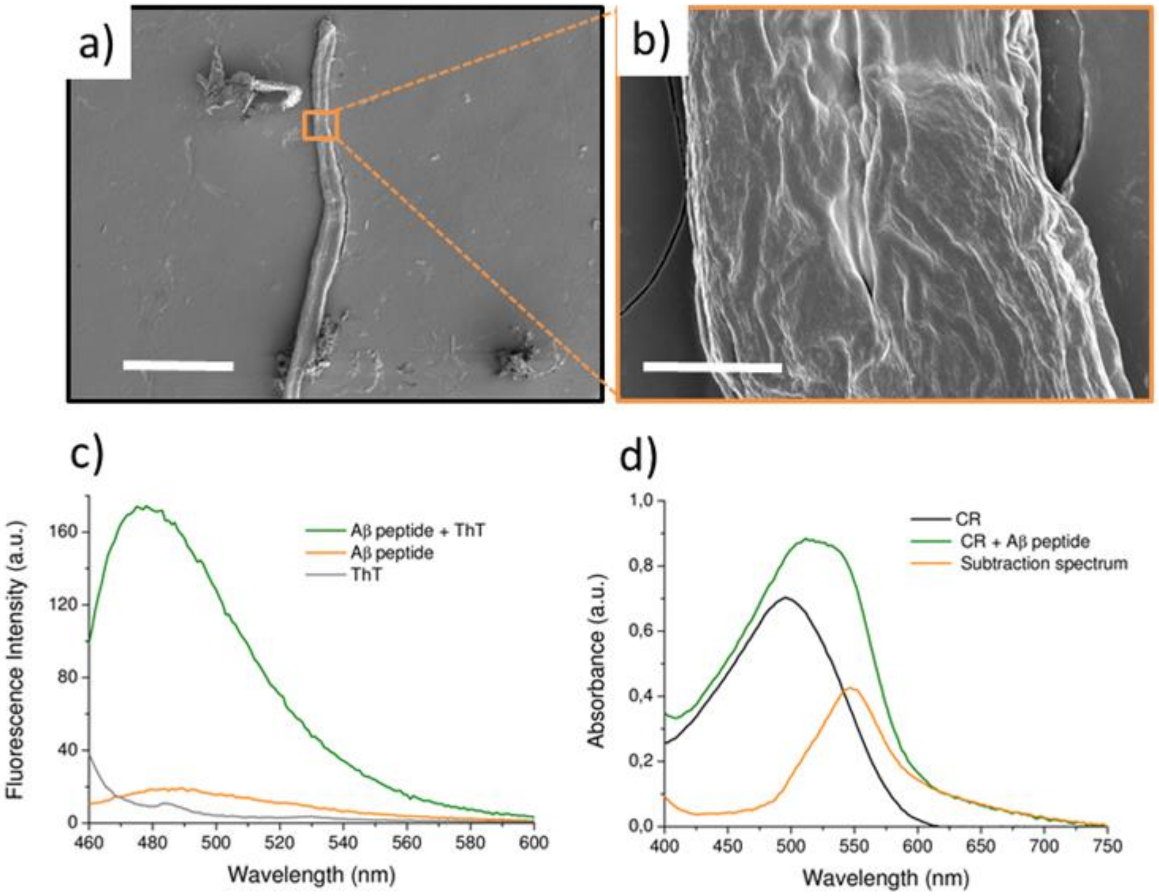

Figure S1: a) and b) selected micrographs acquired on sample drop-casted on stub by a Aβ-peptide solution at a concentration of 5 mg/mL (scale bar 100 and 5.0 µm, respectively). d) Fluorescence emission spectra of ThT, Aβ-peptide and the mix of ThT and the peptide. e) UV-Vis spectra of Congo Red alone and co-incubated with the Aβ-peptide. Subtraction spectra is also reported.

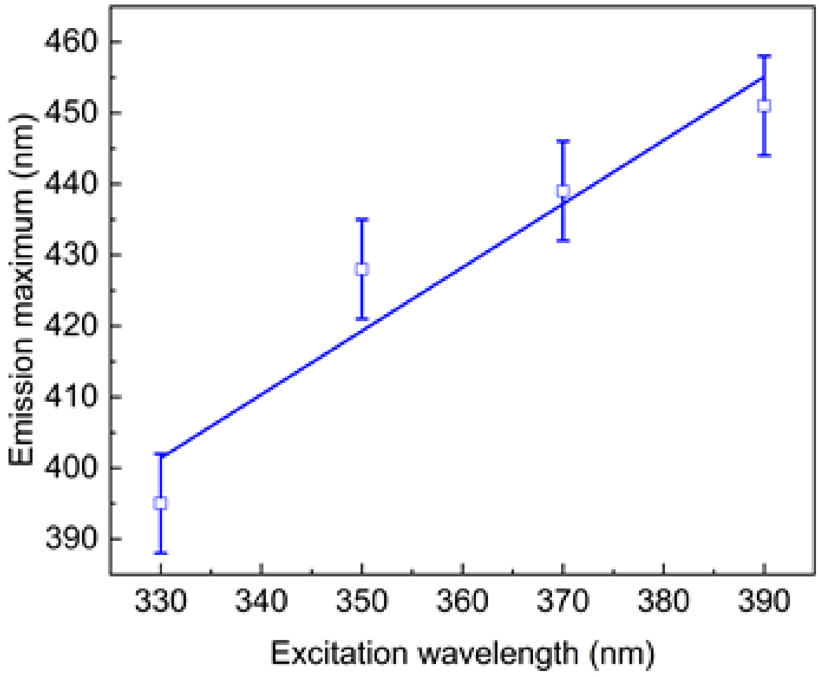

Figure S2: Plots of the maxima positions as function of the excitation wavelength for the H^+-Aβ16-21-O^- peptide film at 100 mg/mL. The linear fit of the data provided slope and intercept values of 0.89 ± 0.18 and 106 ± 67, respectively (Reduced χ^2 = 1.395; Adj. R^2 = 0.882). As can be evidenced, such quantities are fully compatible with those estimated for the same sample at lower concentration (see Figure 2d).

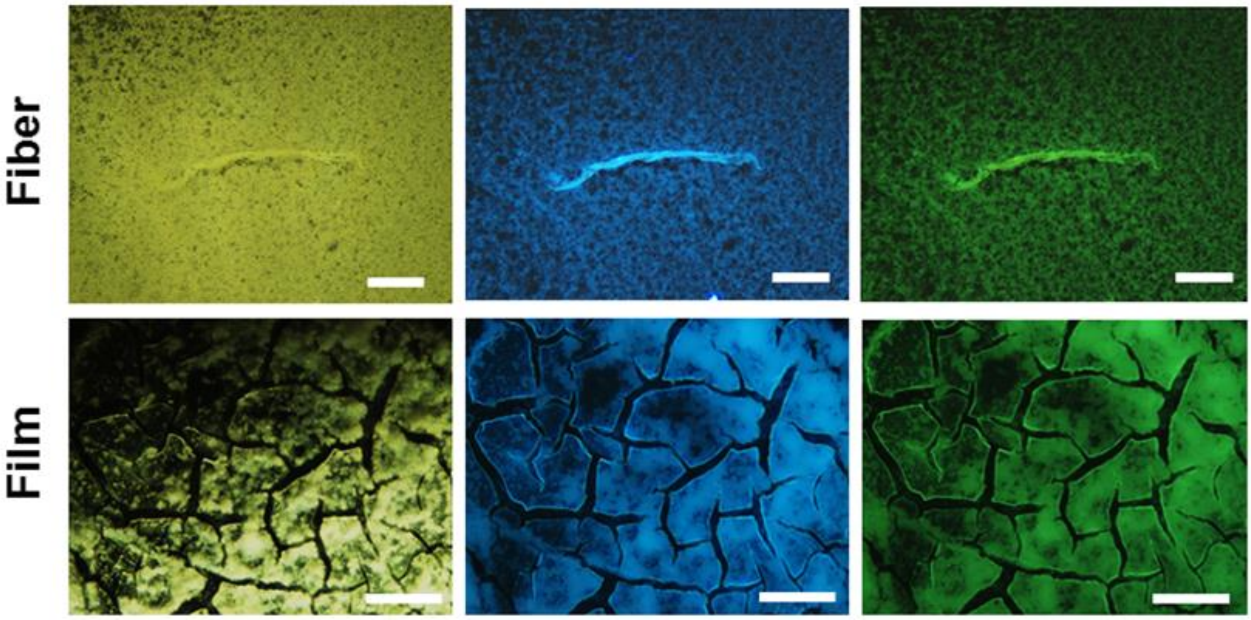

Figure S3: Fluorescence microscopy images of H^{+}-F6-O^{-} fiber and film. Samples were prepared by deposition of peptide solutions 5.0 and 50 mg/mL in HFIP on a clean coverslip glass and slowly dried at room temperature. On the left, images in the bright field, in the center and on the right fluorescence images in the DAPI (4’,6-diamidino-2-phenylindole; λ_{exc} = 359 nm, λ_{em} = 461 nm) and GFP (Green Fluorescent protein λ_{exc} = 488 nm, λ_{em} = 507 nm) spectral regions. Scale bars are 100 and 50 µm for fiber and film, respectively.
